# Real-Time LiDAR 3D Semantic Segmentation via Multi-View and Cross-Modal Compact Featuring Two-Branch Knowledge Distillation

**DOI:** 10.3390/s26061860

**Published:** 2026-03-15

**Authors:** Yun Zhang, Kun Qian, Zihan Zhang, Min’ao Zhang, Hai Yu

**Affiliations:** 1 School of Automation, Southeast University, Nanjing 210096, China; 2 Southeast University Shenzhen Research Institute, Shenzhen 518063, China; 3 Information and Communication Research Institute, China Electric Power Research Institute Co., Ltd., Nanjing 210000, China

**Keywords:** LiDAR, semantic segmentation, multi-view and cross-model fusion, knowledge distillation, handheld 3D scanner

## Abstract

Simultaneous online mapping and semantic segmentation using handheld scanners supports various environmental inspection and measurement tasks. For such scanners, combing visual and LiDAR data is beneficial for improving the segmentation performance. But the direct fusion of multi-modal and multi-view features faces challenges in terms of both real-time performance and robustness. To address these challenges, this paper proposes a multi-view and cross-modal knowledge distillation method for supporting runtime LiDAR-only semantic segmentation. The proposed method hierarchically compacts multi-view and cross-model priors and distills them into two branches to improve segmentation accuracy. In addition, we design an improved data augmentation technique based on PolarMix for rendering more realistic point cloud scenes. The experimental results on the SemanticKITTI and nuScenes datasets demonstrate that the mIoU of our approach outperforms the state-of-the-art knowledge-distillation-based methods. In addition, mapping experiments using a handheld scanner demonstrate the proposed method’s superior real-time performance and accuracy.

## 1. Introduction

**Figure 1:**
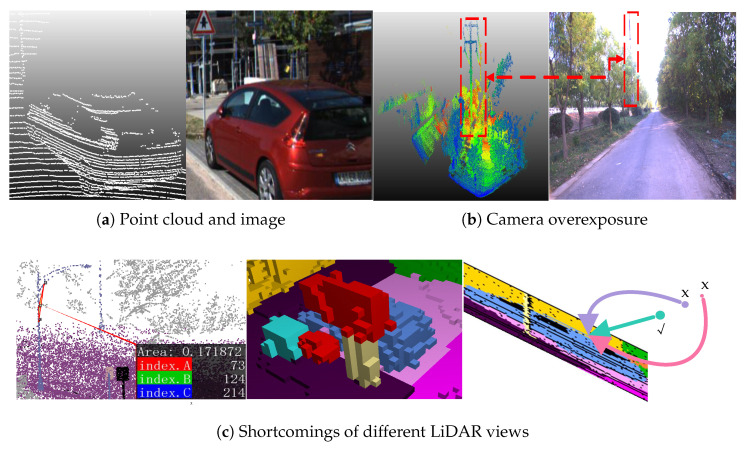
Visualization Multi-view and Cross-modal sensor data characteristics: (**a**) accurate position of the point clouds, as well as color and texture of the image, (**b**) one drawback of the camera: overexposure, (**c**) shortcomings of different LiDAR views: disorder, quantization error, projection error. x indicates that this point is obscured in the point cloud projection image.

Handheld LiDAR scanners are increasingly being used in inspection applications. The Online semantic segmentation of point clouds from such scanners during the scanning process enables fine-grained and visible measurement tasks [1,2], such as measuring the height of trees and poles. For semantic segmentation tasks, cameras can provide color and texture features of the surrounding environment, thereby achieving high segmentation accuracy [3]. However, cameras are inherently susceptible to changes in lighting conditions (as shown in Figure 1a) and have difficulty obtaining precise depth information for distant objects. In contrast, the Light Detection and Ranging (LiDAR) sensor offers precise spatial data over a wide range and is robust to illumination fluctuation. With the rapid development of deep learning, point cloud semantic segmentation technology has made significant progress. However, the performance of deep learning models is highly dependent on the scale and diversity of the training data. In practical applications, point cloud data is constrained by factors such as sensor performance, scanning range, and environmental occlusion, and it often suffers from uneven point density and sparsity, which make the model prone to overfitting and limits its generalization. Furthermore, the limitation of LiDAR is that it cannot capture information such as color and texture (as shown in Figure 1b), and the sparsity and disorder of the large number of point clouds also pose challenges to its semantic segmentation methods.

Data augmentation techniques have become an important approach to addressing data distribution issues in LiDAR semantic segmentation. By applying a series of transformation operations to the original data, they artificially expand the size and diversity of the training set, thereby enhancing the model’s robustness and generalization performance. Among the point cloud data augmentation methods, PolarMix [4] is a representative advanced enhancement algorithm. This method draws on the idea of image mixture enhancement and performs multi-angle rotations and pasting operations on point cloud instances. It effectively enriches the distribution of point cloud data while preserving the scene’s geometric structure. However, the PolarMix method has some limitations: it applies the same rotation-pasting operations to all instance classes, regardless of differences in the number of instances in each category in the original data set. This strategy fails to fully account for the significant class imbalance in the original point cloud data. Moreover, this strategy does not account for the physical distribution characteristics of real point clouds and introduces a large number of mutually occluded, penetrating, or suspended objects. This problem limits the model’s ability to learn from sparse classes, in turn affecting overall segmentation performance. To address the issue of the limited variety of LiDAR features, many approaches utilizing different views of LiDAR have emerged in recent years, primarily encompassing point-based methods [5,6], voxel-based methods [7,8], and projection-based methods [9,10]. However, each view has its limitations. For instance, as illustrated in Figure 1c, the inherent disorder of the point cloud, the quantization error caused by voxelization, and the error resulting from projection all impose constraints on accuracy. To address the issues mentioned above, the multi-view fusion strategy takes advantage of the collaborative strengths of multiple views, achieving more accurate segmentation, as exemplified in [11]. However, these approaches often encounter redundancy features between different views originating from the same sensor, and the lack of feature diversity also constrains segmentation accuracy.

Compared with multi-view methods, multi-modal methods that fuse more types of sensors can bring more diverse features and theoretically improve segmentation accuracy. However, they also face two challenges: degradation in accuracy and inherently low real-time performance when applied to a handheld 3D scanner. For example, the calibration errors between multiple sensors could potentially reduce the segmentation accuracy. And abnormal data from any sensors, such as the overexposure shown in Figure 1b, is detrimental to accuracy and robustness. Additionally, whether for multi-view methods or multi-modal methods, they typically require dedicated feature encoders for each input data. These encoders impose a significant computational load and consume valuable memory resources, resulting in a decrease in real-time performance and hindering the scalability of the data. These challenges mentioned above make it difficult to combine online semantic segmentation with other high-real-time and highly accurate tasks on handheld 3D scanners.

To address the issues above, this paper proposes a two-branch feature enhancement framework that utilizes multi-modal and multi-view feature fusion to strengthen a LiDAR-only semantic segmentation baseline. The lightweight baseline in charge of inference constitutes the main branch, which is instantiated by a voxel-based lightweight segmentation paradigm in this paper. To compensate for the accuracy loss caused by lightweighting, the auxiliary branch hierarchically compacts the input multi-view and multi-modal features in a mutual-attention-based manner, followed by distilling the final fused features to all branches. In contrast to the lightweight main branch, which prioritizes high real-time performance, the lightweight auxiliary branch is designed to improve network scalability by conserving memory resources as much as possible. To further improve accuracy, we also propose an enhanced data augmentation method called APolarMix, which is based on the classical PolarMix method. In contrast to the original vision, which employs scene-level swapping by exchanging sectors of LiDAR scans and instance-level holistic rotate-pasting, the primary objective of APolarMix is to render the inserted object point clouds more realistic.

In summary, the main contributions of this paper are as follows:

- We propose a multi-view and cross-modal fusion method for improving accuracy while maintaining the real-time performance of semantic segmentation. The key to this framework lies in creating compact features through a mutual-attention-based three-step gated mechanism (GM) fusion across multi-view and cross-modality features. This design also offers low coupling between input features and easily allows for the addition of more view/modality features at a low cost.
- We adopt bidirectional intra-channel enhancement and inter-channel fusion in the auxiliary branch combined with two-branch knowledge distillation (KD) to strengthen both branches simultaneously. By improving the performance of the auxiliary branch, the main branch’s performance can also be enhanced indirectly. In contrast to full-modal and full-view inference, our approach significantly enhances segmentation accuracy.
- An enhanced PolarMix-based data augmentation algorithm is proposed. This method is designed to achieve a more realistic augmentation of point clouds by incorporating rotational adjustments, height alterations, and occlusion rectifications.
- Compared to other KD-based point cloud semantic segmentation methods, our method has outperformed the state-of-the-art performance in both mIoU and inference speed on the SemanticKITTI and the nuScenes datasets. Additional mapping experiments further demonstrate the necessity of real-time and accurate semantic segmentation for handheld applications.

## 2. Related Works

### 2.1. LiDAR Point Cloud Semantic Segmentation

Current LiDAR-based semantic segmentation technology routes can be predominantly classified into three categories: point-, voxel-, and projection-based methods.

Point-based methods, such as PointNet++ [5], are adept at processing 3D point clouds. These techniques exhibit considerable efficacy when applied to small point cloud datasets. However, when dealing with large-scale point clouds, their accuracy will decrease. To address this challenge and improve performance on large-scale scenes, RandLA-Net [6] has been proposed. This network introduces simple yet effective random down-sampling procedures with local feature aggregation strategies.

Projection-based methods are designed to map various perspectives, such as a bird’s-eye view (BEV) [12] or range view (RV), of 3D point clouds onto a 2D image plane. These approaches frequently attain high real-time performance. Nevertheless, they are susceptible to information loss due to occlusions. Ref. [9] introduces a rapid, GPU-accelerated k-nearest neighbor (KNN) search algorithm to obtain a collective vote of the nearest k points for each point. An end-to-end method [13] circumvents this post-processing challenge by introducing KPConv layers before the final classification stage.

Some voxel-based baselines [14] attempt to achieve faster 3D convolution and enhance performance while reducing computational requirements. On the other hand, others employ different convolution shapes and network structures to enhance feature capture capabilities. Ref. [8] introduces an asymmetrical residual block designed to diminish computational complexity while preserving features pertinent to cuboid-like objects. Furthermore, AF2S3Net [15] proposes two innovative attention blocks that effectively capture local and global contextual information.

### 2.2. Cross-Modal and Multi-View Semantic Segmentation

Cross-modal and multi-view methods exhibit many configurations. The fusion methods of point and voxel have been explored by several researchers, including [16,17]. Specifically, ref. [16] introduces a semantic segmentation framework for single-sweep LiDAR point clouds, which capitalizes on contextual shape priors derived from a semantic scene completion network. Ref. [17] combines the multi-layer perceptron with the sparse convolution in a block to facilitate learning robust representations. The work by [18] employs RV and BEV data for point cloud semantic segmentation. It presents a novel module that utilizes geometric relationships within an end-to-end learning paradigm to align and disseminate complementary information across disparate views in a bi-directional manner. Ref. [19] projects point features into 2D space and combines them with range data. Ref. [20] achieves multi-scale feature extraction by fusing range information, point coordinate projection, residual projection, and intensity projection. Ref. [11] proposes a deep fusion framework that incorporates multi-level mutual information interactions across point, voxel, and range views, and it utilizes a gated fusion module to fuse all features adaptively.

### 2.3. Knowledge Distillation

Knowledge distillation was first introduced to help compress a large-scale teacher network into a smaller student network [21]. In the field of computer vision, some KD-based methods utilize various forms of features to distill knowledge, such as visual attention map distillation [22], mutual learning distillation [23], and mutual relation distillation [24]. In the context of point cloud data, some methods extract prior information from an image. For instance, ref. [25] enables voxel-based semantic segmentation networks to assimilate partial image features.

## 3. Method

**Figure 2:**
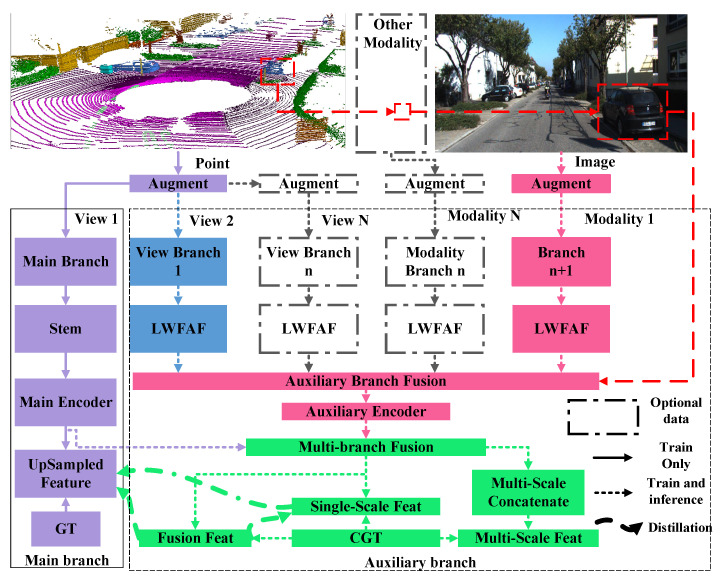
The overall structure of the framework. The main branch takes one LiDAR view as input (depicted in purple). The auxiliary branch fuses features from different LiDAR views and cross-modal data from various sensors, and it distills these fused features to itself and the main branch.

**Figure 3:**
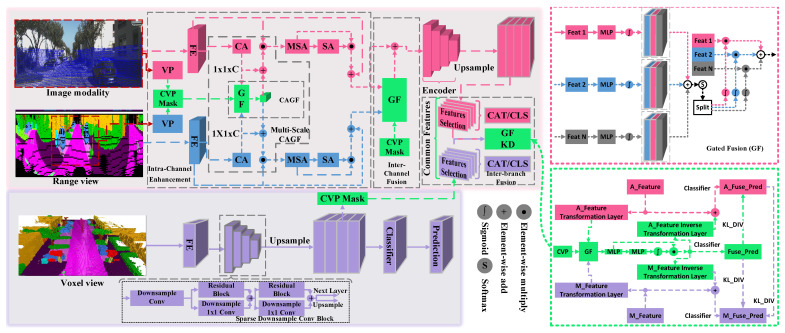
Details of a framework implementation. The main branch (voxel) is a simplified semantic segmentation scheme, shown by solid lines. The auxiliary branch first extracts features from the range and the image and then enhances them using the first-level GM. Next, the second-level fused features are encoded to generate multi-scale features. Finally, the third-level GM fusion and two-branch knowledge distillation are carried out in the inter-branch fusion scheme. The entire auxiliary branch is shown with dotted lines, indicating that it is only active during training.

In this section, we will introduce our method. Firstly, the simplified structure of the proposed scalable network is shown in Figure 2. The network architecture comprises a main branch that leverages features from the LIDAR sensor and an auxiliary branch designed for modality (from other sensors/features)/view (from LiDAR) fusion. The core idea of this scalable network is to generate compact features through hierarchical feature fusion, which utilizes a three-step GM fusion method based on mutual attention and a multi-branch knowledge distillation (KD) strategy. The GM-based three-step fusion encompasses stages of light-weighting feature augmentation and fusion (LWFAF), auxiliary branch fusion, and multi-branch fusion. When fusing more modalities or views, we only need to add the corresponding LWFAF module to the network, thereby enabling the network to achieve excellent scalability. The auxiliary branch fusion aims to fuse and compact the output features of all LWFAF modules. This strategy allows for a single encoder to extract multi-view, cross-modal, and multi-layer features. The third level of gated fusion further fuses the encoded features from the main and the auxiliary branches at each layer and employs KD to enhance the network’s segmentation accuracy. Figure 3 presents a detailed implementation of the framework, which utilizes the voxel view (V) as the main branch input while adopting the range view (R) and image modality (I) as auxiliary branch inputs. Section 3.1 describes the main branch, which forms the network’s baseline and is involved in both training and inference. This baseline balances real-time performance with accuracy. Section 3.2 and Section 3.3 detail the structure of the auxiliary branch. Section 3.2 emphasizes the internal multi-level fusion mechanisms, while Section 3.3 discusses the fusion between branches. It is important to note that the auxiliary branch is only used during training.

### 3.1. Structures of the Main Branch

In the main branch, for point clouds $P={\left\{\left(\begin{array}{ccc} x_{i} & y_{i} & z_{i} \end{array}\right)\right\}}_{i}^{N}$, we first calculate a point-to-voxel mapping of the l-th layer through (1) and record a coordinate-based hash mapping used for voxel-to-point mapping.

(1) $$\begin{array}{c} D_{l}^{voxel}={\left(\begin{array}{ccc} \left\lfloor x_{i}/r_{l}\right\rfloor & \left\lfloor y_{i}/r_{l}\right\rfloor & \left\lfloor z_{i}/r_{l}\right\rfloor \end{array}\right)}_{i}^{N}\in R^{N*3} \end{array}$$

where *N* is the number of point clouds, and each point has three channels: x,y,z. $r_{l}$ is the voxelization spatial size in the *l*-th layer, and ⌊⌋ is the floor operation. Meanwhile, as shown in Figure 3, we use sparse convolution to extract voxel features through the feature extraction head (FE). Subsequently, an encoder is employed to extract features with different receptive fields in a hierarchical manner. To facilitate subsequent feature fusion, we upsample the features to their original spatial resolution. Finally, a simple MLP-based classifier is employed to categorize the concatenated multi-layer features.

### 3.2. Gating-Mechanism-Based Hierarchical Feature Fusion

In the auxiliary branch, the first step is deriving valid projection points (VPs) through projection relationships that map point clouds to other representations. Specifically, for the range view, the projection of point clouds is mathematically expressed by Equation (2)

(2) $$\begin{array}{ccc} \; & {\left(\begin{array}{cc} u & v \end{array}\right)}^{T}=\{\frac{1}{2}\left[1-\frac{\arctan\left(y,x\right)}{\pi }\right]W_{r}, & \left[1-\frac{\arcsin\left(zr^{-1}\right)+f_{up}}{f}\right]H_{r}\} \end{array}$$

where $H_{r}$ and $W_{r}$ are range image size, $\left(\begin{array}{cc} u & v \end{array}\right)$ is the pixel coordinate, $f=f_{up}+f_{down}$, is the vertical field of view of the sensor, and $r={∥p_{i}∥}_{2}$ is the depth of each point. When multiple points project onto the same pixel, we choose the point with the smallest depth as the projection point, ensuring that all points depicted in the range view are encompassed within the sensor’s current field of view. Furthermore, the spatial projection from the LiDAR to the camera is delineated by Equation (3)

(3) $${\left(\begin{array}{ccc} u_{i} & v_{i} & 1 \end{array}\right)}^{T}=\left\lfloor K\times T/z_{i}\times {\left(\begin{array}{cccc} x_{i} & y_{i} & z_{i} & 1 \end{array}\right)}^{T}\right\rfloor$$

where $K\in R^{3\times 4}$ and $T\in R^{4\times 4}$ are the camera intrinsic and extrinsic matrices. Furthermore, we can obtain the common projection points (CVP) by combining on all mapping relationships (2) and (3).

Meanwhile, the FE (2D convolutions for RGB image and proximity convolution [26] for range image) is utilized to extract features from each input data. To fully leverage these features, the auxiliary branch further extracts weighted features based on channel attention, including the extraction of channel attention weight (CA) and the gated fusion of CA (CAGF). The GF module first computes mutual attention weights and splits them into N sub-weights for subsequent weighting. As illustrated in Figure 3, multi-scale features can be obtained by stacking multiple CAGF layers and aggregated by a multi-scale aggregation layer (MSA). Finally, inspired by CBAM [27], we employ spatial attention (SA) to enhance the features further.

Traditional multi-view and cross-modal deep fusion networks usually assign an encoder for each input data. However, because of limited computing resources, the high computational load caused by larger features and parameters makes these methods less suitable for fusing additional views and modalities. Therefore, the proposed approach uses an inter-channel GF layer to fuse previous features in the auxiliary branch and then employs a single encoder to extract multi-receptive field features. This method ensures that adding more modalities or views does not significantly increase the computational load.

### 3.3. Inter-Branch Fusion

To enhance and distill features more effectively while preserving the information from different receptive fields, the features (M_Feature) of each main branch encoder layer are further fused with the corresponding auxiliary encoder features (A_Feature). As shown in Figure 3, the third-level gated fusion (inter-branch fusion) occurs on the features of each encoder layer. Because of the different representations between features, feature transformation layers are employed before fusion to bridge the feature gap in both directions. After the gated fusion, feature inverse transformation layers are also applied to bridge the gap between the fused features and the original encoder features. The inverse-transformed features are then aggregated with their original features and input into a classifier for prediction. The fused features are distilled to both branches. Additionally, the auxiliary branch predictions are also distilled to the main branch. This two-branch distillation strategy aims to improve the segmentation accuracy of both branches at the same time. By enhancing the auxiliary branch’s performance, we also strengthen the main branch’s segmentation performance.

### 3.4. Augmented PolarMix

**Figure 4:**
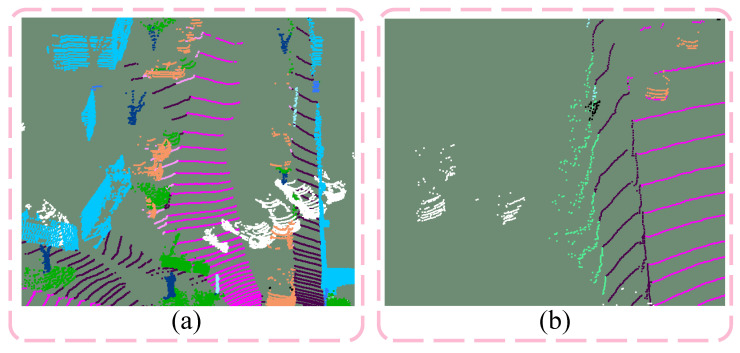
An enhanced point cloud frame by PolarMix, where the white points represent inserted instance objects and other colors indicate different categories of objects in the original scene. (**a**) illustrates an example of the interpenetration problem, where inserted cars overlap with the scene’s walls (blue points), and (**b**) shows a case of objects incorrectly placed outside the scene without azimuth correction.

The PolarMix mainly enhances point cloud data by replicating the entire instance instead of adding objects individually. It also does not fully account for how adaptable the objects are when integrated into the scene, which can lead to floating objects, collisions, and mutual occlusion, as illustrated in Figure 4a. To enhance the realism of the augmented point cloud, this paper introduces the APolarMix data augmentation technique. This approach includes two parts: point cloud preprocessing and point cloud augmentation.

#### 3.4.1. Point Cloud Preprocessing

Point cloud preprocessing is a step carried out offline before the network training stage. Point cloud preprocessing involves three main steps. First, we extract ground points (such as points on the road and parking), segment individual objects, and cluster unlabeled objects. Next, we calculate the distances from the center of each object to the ground plane and the LIDAR sensor. Finally, we create bounding boxes for each object and remove the ground points beneath these boxes.

#### 3.4.2. Point Cloud Augmentation

**Figure 5:**
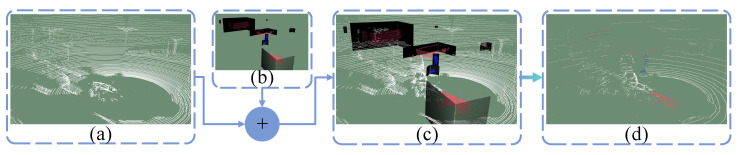
(**a**) Original scene. (**b**) Inserting objects with generated walls. (**c**) Fusion of the original scene and the inserted instances. (**d**) Eliminating occluded points through projection and back projection.

This data augmentation method is only used during training, so it will not affect the inference time. For the current point cloud scene, $P^{a}$, and a randomly chosen scene, $P^{b}$, we first use PolarMix for initial data augmentation. It is important to note that any objects whose bounding boxes overlap with the scene’s point cloud must be removed during the instance-level rotate-paste step. Next, we locate the corresponding ground point clouds of $P^{a}$ and $P^{b}$ within the ground point cloud set, *G*, and perform swapping and downsampling to create a temporary ground point cloud, $P_{g}^{temp}$. Then, a ground point in $P_{g}^{temp}$ is randomly selected, and the distance $D_{g}^{temp}$ from this point to LIDAR is calculated. In the instance set *I*, objects are randomly selected based on weights, classification cls, and the distance $D_{g}^{temp}$. This distance constraint ensures that the sparsity of these objects matches real-world conditions. Every time an object is inserted, elevation and azimuth are calibrated to ensure contact with the ground. In particular, using the ground point as a reference for azimuth calibration prevents objects from exceeding scene boundaries. Figure 4b illustrates a case without azimuth correction. The ground points beneath the bounding box of each inserted object are removed from $P_{g}^{temp}$. As shown in Figure 5, to improve scene realism [28], we generate a point cloud wall for each instance object in a direction far from the LIDAR and perform scene-wise range projection and back projection. This process eliminates the point clouds obscured by the inserted instance objects.

## 4. Experiments

### 4.1. Experimental Setup

#### 4.1.1. Dateset

**Figure 6:**
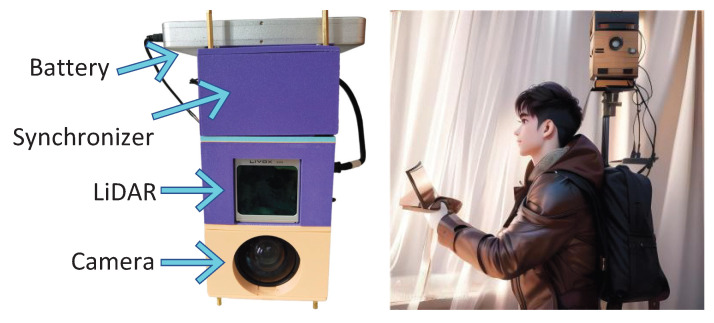
Handheld/backpack sensors platform.

The SemanticKITTI [29] comprises 43,551 LiDAR frames, with each frame containing approximately 130,000 points. The nuScenes dataset [30] includes 1.4 billion labeled points within 850 scenes for training and validation and 150 scenes for testing. The custom dataset was collected using a handheld/backpack scanner platform (as shown in Figure 6). This scanner includes a Livox Avia LiDAR (Livox Technology Co. Ltd., Shenzhen, China), a camera, a battery, and a synchronizer to trigger all sensors. The collection rates for point clouds and RGB images were set to 10 Hz, and the IMU data was sampled at 200 Hz. In order to prevent the degradation of multi-modal fusion accuracy caused by extreme lighting conditions during the training of the network, we collected data during the daytime, when the illumination was relatively stable, avoiding direct camera angles towards the light sources, which could lead to overexposure. Additionally, we enable the automatic gain control and auto-exposure to compensate for the changing light conditions.

#### 4.1.2. Network Implementation

In this study, the encoder of the main branch is based on the architecture of SPVCNN, and the sparse convolution operation is based on the TorchSparse++ library [31]. The layer dimensions of the encoder are 16, 32, 64, and 128. Subsequently, an MLP is used as a decoder to predict the classification on the upsampled features.

For the auxiliary branch, the FEs of the RGB image and the range image use a three-layer 3 × 3 convolution to learn local features. The only difference for the FE of the range image is that the first convolution layer is replaced by proximity convolution. The auxiliary branch directly employs the encoder of ResNet34 [32]. Before feature fusion in the inter-branch scheme, the output feature dimension of each encoder’s layer is then transformed to a unified 64 dimensions. The initial voxel size is 0.05 for SemanticKITTI and 0.1 for nuScenes. The RGB image and the range image are resized to 640 × 320 and 64 × 2048. We apply the cross-entropy loss and Lovász loss [33] as the loss functions. The batch size, learning rate, and number of epochs are 2, 0.24, and 80, respectively. The network is trained using an SGD optimizer. To evaluate real-time inference performance, the frames per second (FPS) are measured on a notebook computer equipped with an Intel Core i7-11800H CPU and an NVIDIA GeForce RTX 3070 GPU.

#### 4.1.3. Evaluation Metrics

In this paper, we adopt a common evaluation metric, mIoU (mean intersection over union). The mIoU metric can be defined as:

(4) $$\begin{array}{c} mIoU=\frac{1}{C}\sum_{c=1}^{C}\frac{TP_{c}}{TP_{c}+FP_{c}+FN_{c}} \end{array}$$

where $TP_{c}$, $FP_{c}$ and $FN_{c}$ represent true positive, false positive, and false negative predictions for the given class, *c*, respectively, and *C* is the number of classes.

### 4.2. Sensors Calib

Since the position and orientation of the handheld device are not fixed, it is necessary to consider minimizing the projection error of the LiDAR point cloud onto the image. This error is divided into two parts. The first part is the static error, which is calibrated by collecting calibration plates from near to far for the LiDAR and the camera. To account for motion-induced dynamic errors, we project all points in each point cloud frame onto the coordinate system of the first point using their timestamps and IMU data.

### 4.3. Results on SemanticKITTI and nuScenes

In Table 1 and Table 2, ‘VIR’ represents the combination of voxel, RGB image, and range image, while ‘LC’ refers to LiDAR and camera. These tables demonstrate that voxel-based methods generally outperform range image and point cloud approaches. Furthermore, multi-view methods show significant advantages over single-view counterparts; for example, RPVNet exceeds SPVNAS in segmentation accuracy, and similarly, the cross-modal method UniSeg outperforms RPVNet. For KD-based methods, it is also observed that the cross-modal method 2DPASS outperforms the unimodal PVKD, and our method surpasses 2DPASS. The mIoU of UniSeg (147.6 M) surpasses our method, which may be due to the fewer parameters and the single-view inference of our approach.

### 4.4. Ablation Studies

The main factors that determine the baseline’s performance are the voxel size and the network scale. Different fusion methods and distillation pathways significantly influence the auxiliary branch, which then indirectly impacts the main branch’s performance. The segmentation accuracy and the computational cost also vary, depending on the combination of views or modalities used. Additionally, to further validate the APolarMix, a comparison with PolarMix is necessary.

#### 4.4.1. Comparisons of Different View-Modality Combinations and Voxel Sizes

As demonstrated in Table 3, both the segmentation accuracy and the inference time decline as the voxel size increases. For the VIR mode, the mIoU scores for different voxel sizes are 69.91 (considered as 100.0%), 69.85 (99.7%), and 68.92 (98.4%), while the corresponding inference times are 310 s (considered as 100%), 205 s (66.1%), and 132 s (42.6%). Compared to the slight drop in segmentation accuracy, the significant boost in the inference speed is clear. This paper chooses a voxel size of 0.05 to balance segmentation accuracy with real-time performance.

#### 4.4.2. Comparisons of Different View-Modality Combinations and Parameter Ratios (PRs)

The dimensions of each layer of the main branch encoder are 16, 32, 64, and 128. Consequently, this paper compares the network’s performance with larger dimensions, as shown in Table 4. When the number of parameters is doubled, the mIoU of the VIR mode reaches 69.85 at 100% PR (considered 100%), and at 200% PR, the mIoU reaches 69.98 (100.18%). However, the inference time increases from 205 s (considered as 100%) to 321 s (156.59%). Therefore, this work selects a smaller set of parameters to enhance system real-time performance without a significant loss in accuracy.

#### 4.4.3. Comparisons of Different Fusion Methods in the Auxiliary Branch

**Figure 7:**
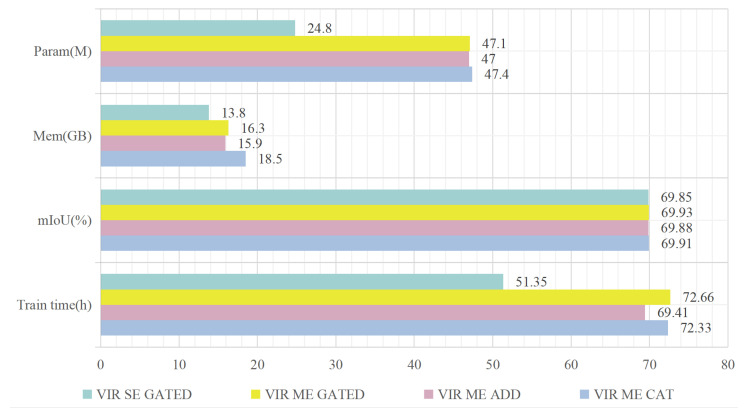
Comparison between single and multiple encoders.

Unlike the traditional method of assigning dedicated encoders to individual input data streams, the auxiliary branch uses a single encoder (SE) to extract multi-receptive-field features from the second-stage gated fusion. To compare these two paradigms, we conducted experiments and present the results in Figure 7. ‘ME’ (multiple encoder) refers to independently assigning encoders for voxel, image, and range. ‘GATED’, ‘ADD’, and ‘CAT’ (concatenate) represent different feature fusion methods. Compared to ‘ME GATED’, the ‘SE GATED’ reduces parameters by 52.7%, the GPU memory usage by 2.5 GB, the mIoU by 0.08, and the training time by 21.31 h. Additionally, compared to ‘ME ADD’ and ‘ME CAT’, the ‘ME GATED’ mode achieves the highest mIoU, with parameters increasing by 0.1 M and decreasing by 0.3 M, memory usage increasing by 0.4 GB and decreasing by 2.2 GB, and training time increasing by 3.25 h and 0.33 h, respectively.

Furthermore, Table 5 shows the changes in the number of parameters, training time, and GPU memory usage across different combinations. Compared to the voxel view, adding another modality or view leads to an apparent increase in both parameters and memory usage. This is mainly due to the extra auxiliary branch. However, when fusing more modalities or views, the number of parameters, GPU memory usage, and training time remain mostly unchanged. This indicates that the proposed network architecture can fuse more views or modalities without significantly raising computational and memory costs.

#### 4.4.4. Comparisons of Different Distillation Methods

The fused features of the third-level GF are distilled into the two branches, and then the auxiliary branch distills the enhanced features into the main branch. In Table 6, the results indicate that distilling fused features into two branches improves segmentation accuracy compared to only distilling features from the auxiliary branch to the main branch. Furthermore, the first and third rows of the table show that distilling fused features to the auxiliary branch can indirectly improve the performance of the main branch.

#### 4.4.5. Evaluation of APolarMix

Table 7 shows a comparison between the original PolarMix and the APolarMix. The latter incorporates several enhancements, including ground point selection, the instance object addition based on weight and distance, elevation/azimuth corrections (E/A cor.), and occlusion point cloud removal based on point cloud wall. The data presented in the table clearly illustrates that these improvements significantly enhance segmentation accuracy. Additionally, we evaluated the average processing time of APolarMix on the SemanticKITTI dataset, with results shown in the “Added Time” column of Table 7. The original PolarMix increases the data loader’s processing time by 0.015 s. Inserting instance objects into the point cloud scene incurs an additional time cost of 0.003 × IN × CN, where IN is the number of inserted instances, and CN is the number of categories. Finally, introducing point-cloud walls to remove occluded points adds 0.02 s to the processing time.

### 4.5. Qualitative Results

**Figure 8:**
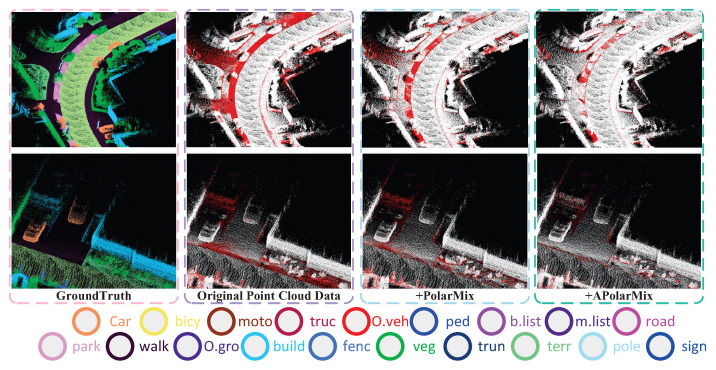
Qualitative results of PolarMix and APolarMix on the SemanticKITTI. The first column shows the semantic ground truth, while the other columns display the segmentation results, with red points indicating incorrect segmentations.

In Figure 8, we compare segmentation errors. The leftmost column shows the ground truth of point clouds, with different colors indicating various semantic objects. The remaining images display inference results of the original point clouds and those enhanced by PolarMix and APolarMix.

**Figure 9:**
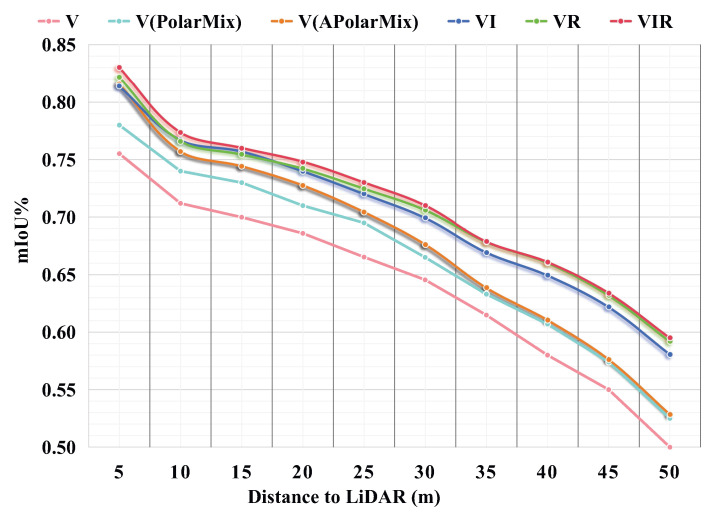
mIoU-distance extended experiment on the SemanticKITTI validation set at different distances.

In Figure 9, we illustrate the segmentation accuracy of the different modality/view combinations and the baseline enhanced by PolarMix/APolarMix at different distances. At close range, APolarMix provides a greater improvement because nearby objects more easily block the point cloud behind them; meanwhile, it is more sensitive to changes in elevation and azimuth angles than distant objects. The VR curve outperforms the VI curve primarily due to the small CVP number of the only forward-facing image in the SemanticKITTI dataset. As a result, the VR curve gets closer to the VIR curve at distances beyond 30 m.

### 4.6. Ground-Constraint-Based Real-Time Mapping

**Figure 10:**
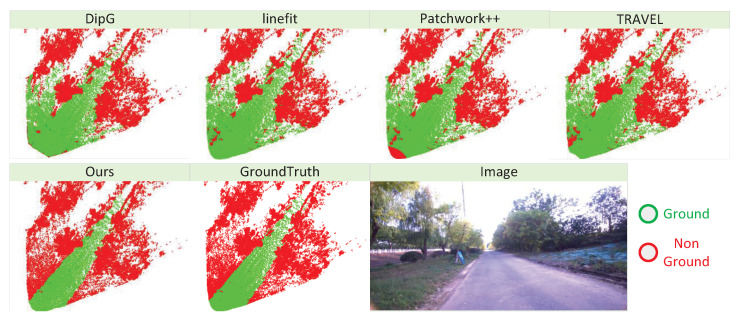
Visualization results of real-time point cloud ground segmentation methods.

**Figure 11:**
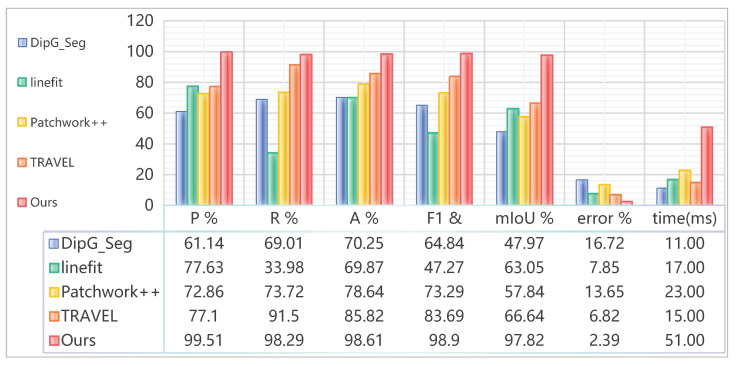
Quantitative results of accuracy of real-time point cloud ground segmentation methods and error of Z-axis vector.

**Figure 12:**
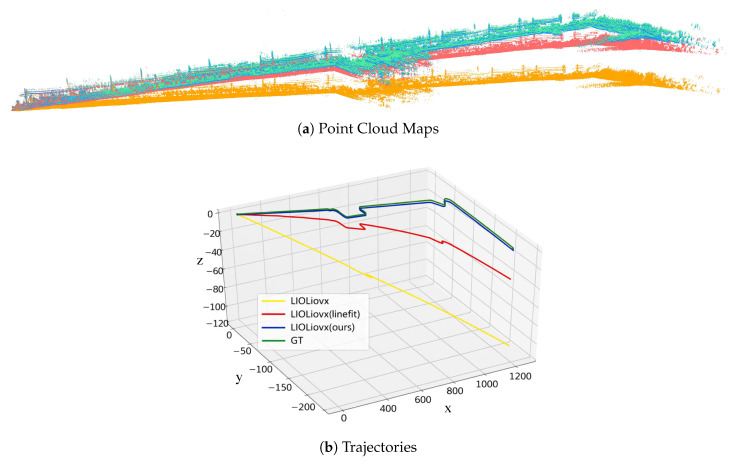
Visualization results of handheld 3D scanning sensor mapping with different pose calibration methods.

The custom dataset inherently has indeterminacy in orientation because the device’s pose is unstable and varies with human movement, causing distortions in the constructed maps. Therefore, real-time pose calibration is essential based on ground constraints. The first step is extracting ground points. Figure 10 shows the visualization results of different ground segmentation methods and ours. And Figure 11 shows the quantitative results: our method achieves the highest precision, recall, accuracy, F1 score, and mIoU. The error metric measures the relative pitch angle error percentage between the calibrated and the uncalibrated sensor data. In Figure 11, the angle error for our methods is the smallest. Although our method requires more computational time, it remains below the LiDAR sampling interval (100 ms), thus meeting real-time requirements. Figure 12 shows the results of maps and trajectories in (a) and (b), the orange result is conducted by LIO-Livox. The red and the blue results are generated by introducing TRAVEL-based pose calibration and our network-based pose calibration. The green points and trajectory are the ground truth.

## 5. Conclusions

This paper proposes a real-time and highly accurate semantic segmentation method through mutual-attention-based three-step GM fusion to obtain compact features and two-branch distilling them for multi-branch collaborative enhancement. These designs endows the network with powerful scalability. Compared to other KD-based high real-time LiDAR semantic segmentation methods, our method further improves accuracy and achieves better mIoU scores on the SemanticKITTI and nuScenes benchmarks. Finally, our APolarMix data augmentation algorithm further improves the segmentation accuracy by generating more realistic point clouds. To further improve accuracy, we plan to explore the use of spatiotemporal information to increase the number of common points within auxiliary branches in future work.

## 6. Discussion

The massive data volume of LiDAR point clouds slows down processing steps such as point cloud processing, segmentation, and mapping. As shown in Table A4, data volume has the greatest impact on mapping speed. Some studies explore how to compress point clouds to reduce data volume. Ref. [39] systematically summarizes common methods for compressing intra-frame and inter-frame point clouds, including but not limited to image-based and octree-based approaches. Although these methods can effectively reduce the size of point cloud data, the errors introduced by decoding compressed point clouds can significantly impact downstream tasks. The octree-based LPCC (LiDAR point cloud compression) method may introduce distortion by merging adjacent points into a single point. In contrast, autoencoder-based methods train encoders and decoders by minimizing reconstruction error, with the decoder essentially predicting the point coordinates. Inaccurate predictions lead to erroneous point representations. Semantically-based LPCC methods can remove points of certain categories from the original point cloud, eliminating unnecessary points before transmission and storage to reduce data size.

Semantic segmentation tasks can serve as both a critical component for point cloud compression and a downstream task. Ref. [40] employs semantic segmentation to separate scene point clouds from object point clouds and then applies distinct compression methods to different types of point clouds. Here, the accuracy of semantic segmentation directly affects the quality of point-cloud compression. Therefore, when deploying such methods in online systems, both segmentation real-time performance and accuracy are important. As one of the downstream tasks in point cloud compression, achieving faster semantic segmentation also relies on reducing data volume and employing more compact feature representations. Consequently, efficient point cloud compression and high-speed semantic segmentation are typically interdependent. Simultaneously achieving efficient compression and semantic segmentation of point clouds represents a key research direction for future downstream tasks.

Additionally, in SLAM systems, LiDAR point-cloud compression may adversely affect localization and mapping performance. For instance, points originating from movable objects can compromise localization accuracy and may be incorrectly displayed in static maps. During inter-frame compression, certain keypoints that are discarded or incorrectly predicted may cause errors in the SLAM system’s inter-frame matching. This leads to pose errors and map distortion. Ensuring map consistency becomes particularly challenging for adjacent frames exhibiting significant differences.

To circumvent the potential degradation in segmentation accuracy caused by point cloud compression, this paper employs multi-modal and multi-view fusion distillation improves the segmentation accuracy of the main branch. Simultaneously, this lightweight network that significantly reduces end-to-end latency, ensuring that the semantic segmentation network does not become a bottleneck for the system’s real-time performance.

## Figures and Tables

**Table 1:** Class-wise mIoU and FPS of Our Method and Other Methods on the SemanticKITTI Leaderboard.

method	mIou (%)	Modality/View	speed (FPS)	car	bicycle	motorcycle	truck	other-vehicle	person	bicyclist	motorcyclist	road	parking	sidewalk	other-ground	building	fence	vegetation	trunk	terrain	pole	traffic sign
RandLA-Net [6]	55.9	P	1.4	94.2	29.8	32.2	43.9	39.1	48.4	47.4	9.4	90.5	61.8	74	24.5	89.7	60.4	83.8	63.6	68.6	51	50.7
RangeNet53++ [9]	52.2	R	13.9	91.4	25.7	34.4	25.7	23	38.3	38.8	4.8	91.8	65	75.2	27.8	87.4	58.6	80.5	55.1	64.6	47.9	55.9
SalsaNext [34]		59.5	36.67	91.9	48.3	38.6	38.9	31.9	60.2	59	19.4	91.7	63.7	75.8	29.1	90.2	64.2	81.8	63.6	66.5	54.3	62.1
PolarNet [12]	54.3	V	7.64	93.8	40.3	30.1	22.9	28.5	43.2	40.2	5.6	90.8	61.7	74.4	21.7	90	61.3	84	65.5	67.8	51.8	57.5
Cylinder3D [8]	68.9		4.76	97.1	67.6	63.8	50.8	58.5	73.7	69.2	48	92.2	65	77	32.3	90.7	66.5	85.6	72.5	69.8	62.4	66.2
AF2S3 [15]	69.7		-	94.5	65.4	86.8	39.2	41.1	80.7	80.4	74.3	91.3	68.8	72.5	53.5	87.9	63.2	70.2	68.5	53.7	61.5	71
SPVNAS [17]	67	Fusion(L)VP	6.92	97.2	50.6	50.4	56.6	58	67.4	67.1	50.3	90.2	67.6	75.4	21.8	91.6	66.9	86.1	73.4	71	64.3	67.3
RPVNet [11]	70.3	Fusion(L)VPR	3.36	97.6	68.4	68.7	44.2	61.1	75.9	74.4	43.4	93.4	70.3	80.7	33.3	93.5	72.1	86.5	75.1	71.7	64.8	61.4
UniSeg [35]	75.2	Fusion(LC)VPRI	-	97.9	71.9	75.2	63.6	74.1	78.9	74.8	60.6	92.6	74	79.5	46.1	93.4	72.7	87.5	76.3	73.1	68.3	68.5
PVKD [36]	71.2	KD(L)V	4.76	97	67.9	69.3	53.5	60.2	75.1	73.5	50.5	91.8	70.9	77.5	41	92.4	69.4	86.5	73.8	71.9	64.9	65.8
2DPASS [25]	72.9	KD(LC)VI	14.33	97	63.6	63.4	61.1	61.5	77.9	81.3	74.1	89.7	67.4	74.7	40	93.5	72.9	86.2	73.9	71	65	70.4
Ours	73.5	KD(LC)VIR	19.8	97.3	70.9	70.3	58.9	67.8	80.1	81.1	66.9	91.2	69.1	77.3	34.1	92.1	69.2	86.5	75.2	71.9	65.8	70.7

**Table 2:** Class-wise mIoU and Frequency-weighted (FW) IoU of Our Method and Other Methods on the nuScenes Leaderboard.

method	mIou (%)	FW mIou (%)	Modality/View	barrier	bicycle	bus	car	construction	motorcycle	pedestrian	traffic cone	trailer	truck	driveable	other flat	sidewalk	terrain	manmade	vegetation
PolarNet [12]	69.4	87.4	V	72.2	16.8	77	86.5	51.1	69.7	64.8	54.1	69.7	63.5	96.6	67.1	77.7	72.1	87.1	84.5
Cylinder3D [8]	77.2	89.9		82.8	29.8	84.3	89.4	63	79.3	77.2	73.4	84.6	69.1	97.7	70.2	80.3	75.5	90.4	87.6
AF2S3 [15]	78.3	88.5		78.9	52.2	89.9	84.2	77.4	74.3	77.3	72	83.9	73.8	97.1	66.5	77.5	74	87.7	86.8
PMF [37]	77	89	KD(LC)RI	82	40	81	88	64	79	80	76	81	67	97	68	78	74	90	88
AMVNet [38]	77.3	90.1	Fusion(L)RB	80.6	32	81.7	88.9	67.1	84.3	76.1	73.5	84.9	67.3	97.5	67.4	79.4	75.5	91.5	88.7
JS3C-Net [16]	73.6	88.1	Fusion(L)PV	80.1	26.2	87.8	84.5	55.2	72.6	71.3	66.3	76.8	71.2	96.8	64.5	76.9	74.1	87.5	86.1
SPVCNN [17]	77.4	89.7		80	30	91.9	90.8	64.7	79	75.6	70.9	81	74.6	97.4	69.2	80	76.1	89.3	87.1
UniSeg [35]	83.5	-	Fusion(L)PVRI	85.9	71.2	92.1	91.6	80.5	88	80.9	76	86.3	76.7	97.7	71.8	80.7	76.7	91.3	88.8
2DPASS [25]	80.8	90.1	KD(LC)VI	81.7	55.3	92	91.8	73.3	86.5	78.5	72.5	84.7	75.5	97.6	69.1	79.9	75.5	90.2	88
Ours	81.7	91.4	KD(LC)VIR	83.2	44.9	95.1	92.4	70.5	88.8	81.5	78.4	87.3	77.6	97.9	68.7	81.1	76.7	92.9	89.8

**Table 3:** Inference Times and mIoU Scores for Various View-Modality Combinations and Voxel Sizes.

PR (%)	Voxel Size (m)	V/M	Infer Time (s)	mIoU (%)
100	0.025	VIR	310	69.91
	0.025	VI		68.65
	0.025	VR		69.02
	0.025	V		65.54
	0.05	VIR	205	69.85
	0.05	VI		68.52
	0.05	VR		68.93
	0.05	V		65.32
	0.1	VIR	132	68.92
	0.1	VI		68.33
	0.1	VR		68.42
	0.1	V		65.16

**Table 4:** Inference Times and mIoU Scores for Various View-Modality Combinations and Parameter Ratios.

Voxel Size (m)	PR (%)	V/M	Infer Time (s)	mIoU (%)
0.05	100	VIR	205	69.85
		VI		68.52
		VR		68.93
		V		65.32
	200	VIR	321	69.98
		VI		68.78
		VR		69.22
		V		66.43

**Table 5:** Number of Parameters, Training Time, and Memory Usage for Different VIR Combinations.

V/M	Param (M)	Epoch Train Time (min)	GPU Mem (GB)
V	2.1	23.9	2.1
V + I	24.9	38.2	13.9
V + R	25	48.6	21.6
V + IR	24.8	38.5	13.8

**Table 6:** mIoU Scores of Different KD Paths.

V/M	KD Pattern			mIoU (%)
	auxiliary -> main	fuse -> main	fuse -> auxiliary	
V	/	/	/	65.32
VIR	√			68.76
		√		69.44
	√		√	68.92
		√	√	69.54
	√	√	√	69.85

**Table 7:** Impact of Different Improvement Methods in APolarMix.

V/M	Data Augmentation Method						mIoU(%)	Added Time
	PolarMix	+	+	+	+	+		
		groundpoint	weight	distance	E/A cor.	wall		
VIR							69.36	/
	√						69.6	+0.015
	√	√					69.67	+0.003 × IN × CN
	√	√	√				69.72	
	√	√	√	√			69.76	
	√	√	√	√	√		69.79	
	√	√	√	√	√	√	69.85	+0.02

**Figure A1:**
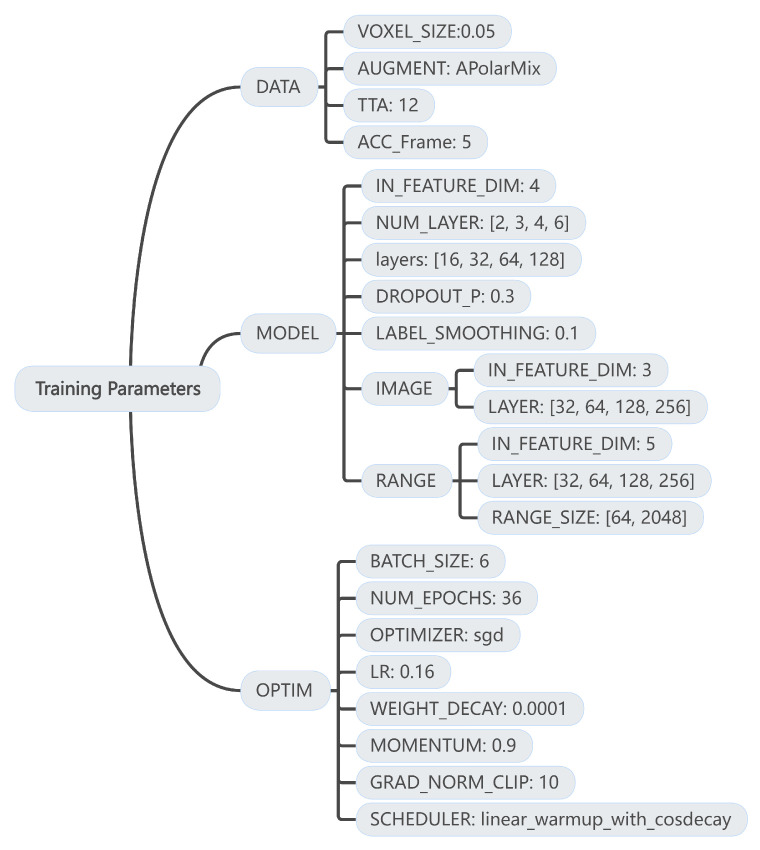
Training parameters.

**Figure A2:**
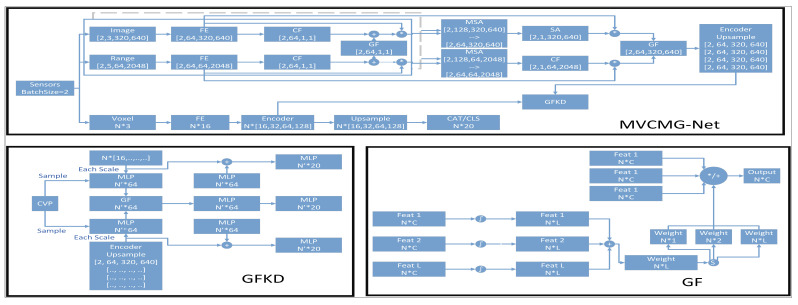
The main structure of network and tensor shape of each stage.

## Data Availability

No new data were created or analyzed in this study. Data sharing is not applicable to this article.

## Appendix A

### Appendix A.1

To facilitate easier reproduction of the network results, we list the key parameters of the network in Figure A1.

### Appendix A.2

We present the gated fusion formula in Equation (A1), where $F_{i}$ is input feature and *i* indicate the indicates which one of the *L* input features this feature refers to. $\bar{F}$ is the fused output features. σ denotes the sigmoid function.

(A1) $$\begin{array}{c} \bar{F}=\sum_{i}^{L}split{\left[softmax\left(\sum_{i}^{L}\sigma \left(MLP\left(F_{i}\right)\right)\right)\right]}_{i}\cdot F_{i} \end{array}$$

The channel/spatial attention weight formulas are shown in Equations (A2) and (A3), where $W_{0}\in R^{(C/2)/C}$ and $W_{1}\in R^{C/(C/2)}$ are MLP weights, *C* is features dimension. $f^{7}\times 7$ represents a convolution operation with the filter size of 7×7. Cat is concatenate operation. $M_{C}\left(F\right)$ and $M_{S}\left(F\right)$ are channel attention map and spatial attention map.

(A2) $$\;M_{C}\left(F\right)=\sigma \left(MLP\left(avgPool(F)\right)+MLP(maxPool(F))\right)\;\;=\sigma (W_{1}(W_{0}(avgPool\left(F)))\;+W_{1}\left(W_{0}\left(maxPool\left(F\right)\right)\right)\right)$$

(A3) $$\begin{array}{c} M_{s}\left(F\right)\;=\;\sigma \left(f^{7\times 7}\left(Cat\left[AvgPool\left(F),maxPool(F\right)\right]\right)\right) \end{array}$$

Equations (A4) and (A5) show sigmoid activation and relu activation, the relu activation is used in encoders and feature transformation. Equation (A6) shows normalization formula.

(A4) $$\begin{array}{c} \mathrm{Sigmoid}\left(x\right)=\sigma \left(x\right)=\frac{1}{1+\exp(-x)} \end{array}$$

(A5) $$\begin{array}{c} \mathrm{ReLU}\left(x\right)={\left(x\right)}^{+}=\max\left(0,x\right) \end{array}$$

(A6) $$\begin{array}{c} y=\frac{x-E[x]}{\sqrt{\mathrm{Var}[x]+\epsilon }}*\gamma +\beta \end{array}$$

### Appendix A.3

Table A1, Table A2 and Table A3 show shapes the network structures. Figure A2 shows the tensor shape.

**Table A1:** The network structure of inter-branch. The arrow indicates the direction of feature distillation.

A_Feature Input (N, 64)		M_Feature Input (N, 64)	
Transformation Layer: Linear (128, 64)/ReLU		Transformation Layer: Linear (128, 64)/ReLU	
GF			
MLP Weighting: Linear (64, 128) ➀ Linear (64, 64)/Sigmoid ➁ ReLU (➀ × ➁)			
Inverse Transformation Layer: Linear (64, 64)/ReLU	Classifier: Linear (20, 64)		Inverse Transformation Layer: Linear 64 × 64/ReLU
ADD/Classifier: Linear (20, 64)	⟵ KL_DIV	KL_DIV ⟶	ADD/Classifier: Linear (20, 64)
KL_DIV ⟶			

**Table A2:** The network structure of GF.

Transformation Feature A (N, 128)	Transformation Feature M (N, 128)
Linear (128, 128)/BN/ReLU Linear (256, 128)	Linear (128, 128)/BN/ReLU Linear (256, 128)
ADD (N, 256)	
Split/Sigmoid (N, 128)	Split/Sigmoid (N, 128)
mul (N, 128)	mul (N, 128)
add (N, 128)	

**Table A3:** The network structure of Sparse Downsample Conv Block.

Downsample SP Conv (Cin, Cout, K = 2, S = 2)/BN/ReLU	
Residual SPConv (Cout, Cout, K = 3)/BN/ReLU SPConv (Cout, Cout, K = 3)	Residual nn.Identity()
ADD/BN/ReLU	
Residual SPConv (Cout, Cout, K = 3)/BN/ReLU SPConv (Cout, Cout, K = 3)	Residual nn.Identity()
ADD/BN/ReLU	
Next Layer	UpSample

### Appendix A.4

We collected 20,000 frames of data through our equipment and measured the end-to-end latency. We recorded the time difference between sensor data publication and calibration completion, the time difference between calibrated data publication and semantic segmentation completion (including voxelization and inference time), and the time difference between semantic label publication and per-frame mapping completion. The results are shown in Table A4. Power measurements comprise two components: sensor power and computer power. Sensors’ power is measured using a Bluetooth-enabled Type-C power meter at a sampling rate of 2 Hz. Similarly, computer power was measured using a Bluetooth-enabled DC power meter at a sampling rate of 1 Hz. All the data represent the average values obtained from multiple long sampling periods. The average computer power consumption was 179 W, while the sensor’s average power consumption was 8.1 W. According to Table A4, the mapping task accounts for most of the end-to-end latency, with the lowest frame rate at 13.97 Hz. However, the data collection, calibration, segmentation, and mapping tasks in this article are carried out in parallel in a pipeline manner; therefore, the power per frame is 13.39 W.

**Table A4:** End-to-end latency test.

Stage	Mean Time Consumption (ms)	Mean FPS	End-to-End Latency (ms)	STD	P95	P99
calib	25.048	39.92334717	154.218	24.44	183.3	187.3
voxelization	16.3	17.36111111				
inference	41.3					
mapping	71.57	13.97233478				

## References

[1] Wald J., Tateno K., Sturm J., Navab N., Tombari F. (2018). Real-Time Fully Incremental Scene Understanding on Mobile Platforms. IEEE Robot. Autom. Lett..

[2] Liu H., Liu R., Yang K., Zhang J., Peng K., Stiefelhagen R. HIDA: Towards Holistic Indoor Understanding for the Visually Impaired via Semantic Instance Segmentation with a Wearable Solid-State LiDAR Sensor. Proceedings of the 2021 IEEE/CVF International Conference on Computer Vision Workshops (ICCVW).

[3] Zhu L., Kang Z., Zhou M., Yang X., Wang Z., Cao Z., Ye C. (2022). CMANet: Cross-Modality Attention Network for Indoor-Scene Semantic Segmentation. Sensors.

[4] Xiao A., Huang J., Guan D., Cui K., Lu S., Shao L. (2022). PolarMix: A General Data Augmentation Technique for LiDAR Point Clouds. Proc. Adv. Neural Inf. Process. Syst..

[5] Charles R.Q., Su H., Kaichun M., Guibas L.J. PointNet: Deep Learning on Point Sets for 3D Classification and Segmentation. Proceedings of the 2017 IEEE Conference on Computer Vision and Pattern Recognition (CVPR).

[6] Hu Q., Yang B., Xie L., Rosa S., Guo Y., Wang Z., Trigoni N., Markham A. RandLA-Net: Efficient Semantic Segmentation of Large-Scale Point Clouds. Proceedings of the 2020 IEEE/CVF Conference on Computer Vision and Pattern Recognition (CVPR).

[7] Wang F., Wu Z., Yang Y., Li W., Liu Y., Zhuang Y. (2023). Real-Time Semantic Segmentation of LiDAR Point Clouds on Edge Devices for Unmanned Systems. IEEE Trans. Instrum. Meas..

[8] Zhu X., Zhou H., Wang T., Hong F., Ma Y., Li W., Li H., Lin D. Cylindrical and Asymmetrical 3D Convolution Networks for LiDAR Segmentation. Proceedings of the 2021 IEEE/CVF Conference on Computer Vision and Pattern Recognition (CVPR).

[9] Milioto A., Vizzo I., Behley J., Stachniss C. RangeNet++: Fast and Accurate LiDAR Semantic Segmentation. Proceedings of the 2019 IEEE/RSJ International Conference on Intelligent Robots and Systems (IROS).

[10] Liu Y., Xu L., Hu W., Chen X., Yi B., Mao Q., Kong D., Ruan S. (2024). Accurate 3-D Semantic Segmentation of Point Clouds for Intelligent Vehicles Based on Multiview Edge Guidance and Fusion. IEEE Sens. J..

[11] Xu J., Zhang R., Dou J., Zhu Y., Sun J., Pu S. RPVNet: A Deep and Efficient Range-Point-Voxel Fusion Network for LiDAR Point Cloud Segmentation. Proceedings of the 2021 IEEE/CVF International Conference on Computer Vision (ICCV).

[12] Zhang Y., Zhou Z., David P., Yue X., Xi Z., Gong B., Foroosh H. PolarNet: An Improved Grid Representation for Online LiDAR Point Clouds Semantic Segmentation. Proceedings of the 2020 IEEE/CVF Conference on Computer Vision and Pattern Recognition (CVPR).

[13] Kochanov D., Nejadasl F.K., Booij O. (2020). KPRNet: Improving projection-based LiDAR semantic segmentation. arXiv.

[14] Graham B., Engelcke M., Maaten L.v.d. 3D Semantic Segmentation with Submanifold Sparse Convolutional Networks. Proceedings of the 2018 IEEE/CVF Conference on Computer Vision and Pattern Recognition (CVPR).

[15] Cheng R., Razani R., Taghavi E., Li E., Liu B. (AF)2-S3Net: Attentive Feature Fusion with Adaptive Feature Selection for Sparse Semantic Segmentation Network. Proceedings of the 2021 IEEE/CVF Conference on Computer Vision and Pattern Recognition (CVPR).

[16] Yan X., Gao J., Li J., Zhang R., Li Z., Huang R., Cui S. (2021). Sparse Single Sweep LiDAR Point Cloud Segmentation via Learning Contextual Shape Priors from Scene Completion. Proc. Natl. Conf. Artif. Intell..

[17] Tang H., Liu Z., Zhao S., Lin Y., Lin J., Wang H., Han S. (2020). Searching Efficient 3D Architectures with Sparse Point-Voxel Convolution. European Conference on Computer Visio.

[18] Qiu H., Yu B., Tao D. (2022). GFNet: Geometric Flow Network for 3D Point Cloud Semantic Segmentation. Transact. Mach. Learn. Res..

[19] Alonso I., Riazuelo L., Montesano L., Murillo A.C. (2020). 3D-MiniNet: Learning a 2D Representation From Point Clouds for Fast and Efficient 3D LIDAR Semantic Segmentation. IEEE Robot. Autom. Lett..

[20] Wang S., Zhu J., Zhang R. (2022). Meta-RangeSeg: LiDAR Sequence Semantic Segmentation Using Multiple Feature Aggregation. IEEE Robot. Autom. Lett..

[21] Hinton G.E., Vinyals O., Dean J. (2015). Distilling the Knowledge in a Neural Network. arXiv.

[22] Zagoruyko S., Komodakis N. Paying More Attention to Attention: Improving the Performance of Convolutional Neural Networks via Attention Transfer. Proceedings of the International Conference on Learning Representations.

[23] Li Z., Ming Y., Yang L., Xue J.H. (2021). Mutual-learning sequence-level knowledge distillation for automatic speech recognition. Neurocomputing.

[24] Park W., Kim D., Lu Y., Cho M. Relational Knowledge Distillation. Proceedings of the 2019 IEEE/CVF Conference on Computer Vision and Pattern Recognition (CVPR).

[25] Yan X., Gao J., Zheng C., Zheng C., Zhang R., Cui S., Li Z. (2022). 2DPASS: 2D Priors Assisted Semantic Segmentation on LiDAR Point Clouds. European Conference on Computer Vision.

[26] Sirohi K., Mohan R., Büscher D., Burgard W., Valada A. (2022). EfficientLPS: Efficient LiDAR Panoptic Segmentation. IEEE Trans. Robot..

[27] Woo S., Park J., Lee J.Y., Kweon I.S. CBAM: Convolutional Block Attention Module. Proceedings of the European Conference on Computer Vision (ECCV).

[28] Kim K., Lee S., Kakani V., Li X., Kim H. (2024). Point Cloud Wall Projection for Realistic Road Data Augmentation. Sensors.

[29] Behley J., Garbade M., Milioto A., Quenzel J., Behnke S., Stachniss C., Gall J. SemanticKITTI: A Dataset for Semantic Scene Understanding of LiDAR Sequences. Proceedings of the 2019 IEEE/CVF International Conference on Computer Vision (ICCV).

[30] Caesar H., Bankiti V., Lang A.H., Vora S., Liong V.E., Xu Q., Krishnan A., Pan Y., Baldan G., Beijbom O. nuScenes: A Multimodal Dataset for Autonomous Driving. Proceedings of the 2020 IEEE/CVF Conference on Computer Vision and Pattern Recognition (CVPR).

[31] Tang H., Yang S., Liu Z., Hong K., Yu Z., Li X., Dai G., Wang Y., Han S. TorchSparse++: Efficient Point Cloud Engine. Proceedings of the 2023 IEEE/CVF Conference on Computer Vision and Pattern Recognition Workshops (CVPRW).

[32] He K., Zhang X., Ren S., Sun J. Deep Residual Learning for Image Recognition. Proceedings of the 2016 IEEE Conference on Computer Vision and Pattern Recognition (CVPR).

[33] Berman M., Triki A.R., Blaschko M.B. The Lovasz-Softmax Loss: A Tractable Surrogate for the Optimization of the Intersection-Over-Union Measure in Neural Networks. Proceedings of the 2018 IEEE/CVF Conference on Computer Vision and Pattern Recognition.

[34] Cortinhal T., Tzelepis G., Erdal Aksoy E. (2020). SalsaNext: Fast, Uncertainty-Aware Semantic Segmentation of LiDAR Point Clouds. Advances in Visual Computing.

[35] Liu Y., Chen R., Li X., Kong L., Yang Y., Xia Z., Bai Y., Zhu X., Ma Y., Li Y. UniSeg: A Unified Multi-Modal LiDAR Segmentation Network and the OpenPCSeg Codebase. Proceedings of the 2023 IEEE/CVF International Conference on Computer Vision (ICCV).

[36] Hou Y., Zhu X., Ma Y., Loy C.C., Li Y. Point-to-Voxel Knowledge Distillation for LiDAR Semantic Segmentation. Proceedings of the 2022 IEEE/CVF Conference on Computer Vision and Pattern Recognition (CVPR).

[37] Zhuang Z., Li R., Jia K., Wang Q., Li Y., Tan M. Perception-Aware Multi-Sensor Fusion for 3D LiDAR Semantic Segmentation. Proceedings of the 2021 IEEE/CVF International Conference on Computer Vision (ICCV).

[38] Liong V.E., Nguyen T.N.T., Widjaja S.A., Sharma D., Chong Z.J. (2020). AMVNet: Assertion-based Multi-View Fusion Network for LiDAR Semantic Segmentation. arXiv.

[39] Wang M., Huang R., Xie W., Ma Z., Ma S. (2025). Compression Approaches for LiDAR Point Clouds and Beyond: A Survey. ACM Trans. Multimed. Comput. Commun. Appl..

[40] Wang M., Huang R., Liu Y., Li Y., Xie W. (2025). suLPCC: A Novel LiDAR Point Cloud Compression Framework for Scene Understanding Tasks. IEEE Trans. Ind. Informat..

